# Consequences of Failing to Investigate

**DOI:** 10.3201/eid2505.AC2505

**Published:** 2019-05

**Authors:** Byron Breedlove

**Affiliations:** Centers for Disease Control and Prevention, Atlanta, Georgia, USA

**Keywords:** art science connection, emerging infectious diseases, art and medicine, about the cover, high-consequence pathogens, Ebola virus disease, Nipah virus disease, hantavirus pulmonary syndrome, smallpox, measles, anthrax, Laocöon, Giovanni Battista Foggini, consequences of failing to investigate, viruses

**Figure:**
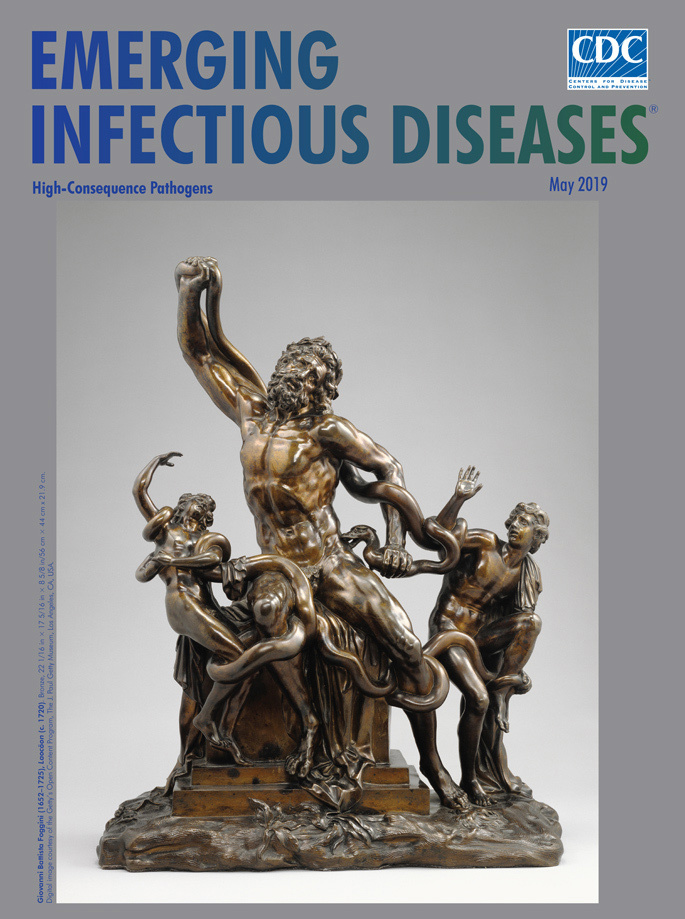
**Giovanni Battista Foggini (1652–1725), Laocöon (c. 1720).** Bronze, 22 1/16 in × 17 5/16 in × 8 5/8 in/56 cm × 44 cm × 21.9 cm. Digital image courtesy of the Getty’s Open Content Program, The J. Paul Getty Museum, Los Angeles, CA, USA

High-consequence pathogens cause diseases such as Ebola virus disease, Rift Valley fever, Nipah virus disease, hantavirus pulmonary syndrome, measles, smallpox, and anthrax. Some such pathogens have the potential to spread rapidly and to cause epidemics. One of the tactics used to control the spread of such pathogens is careful investigation of cases.

Knowing where the danger lies, whether assessing the threat for human infection with high-consequence pathogens or defending a besieged city, is crucial to protecting the health and well-being of the public. Perhaps careful investigation might have changed the outcome of the Trojan War, a mythical protracted, bloody Bronze Age conflict pitting the kingdoms of Troy and Mycenaean Greece against one another.

The Trojan War is one of the most celebrated events in Greek mythology, and many incidents from that epic war have proven to be irresistible subjects for artists, who have reimagined them in sculptures, frescoes, sketches, and paintings. One oft-depicted incident is the ghastly death of the Trojan priest Laocöon and his two sons, Antiphas and Thymbraeus, by serpents.

The reddish-brown bronze statue of Laocöon featured on this month’s cover was fashioned by Italian artist Giovanni Battista Foggini. It is one of a series of small bronzes he created for displaying on tabletops or desks. Foggini used his talent and connections to become the leading court artist and architect to Cosimo III de’ Medici, the Grand Duke of Tuscany. Foggini frequently drew ideas from events portrayed in ancient classical literature. His bronze statue was inspired by the marble sculpture of Laocöon’s death unearthed in Rome in 1506 and later displayed in the Vatican museums.

In this bronze statue, Foggini recreated an event that proved pivotal in ending the war. Following a decade of skirmishes, sieges, counterattacks, and infighting on both sides, the Greek army feigns its withdrawal and leaves a massive, hollow wooden horse near the city’s gates as an offering for the gods. Laocöon, senses treachery and counsels the Trojans either to investigate what is inside the horse or to burn it. When it seems his argument has swayed the majority, the gods intervene and dispatch a pair of sea serpents to silence Laocöon. In his towering epic poem the *Aeneid*, Virgil describes the serpents’ approach:

Looping in giant spirals; the foaming sea Hissed under their motion. And they reached the land,  Their burning eyes suffused with blood and fire,  Their darting tongues licking the hissing mouths.

The *Aeneid* tells us that the serpents “squeezed with scaly pressure” and “fastened their fangs in those poor bodies,” crushing their victims before vanishing with their bodies. Struggling with his foes, the powerful figure of Laocöon dominants the sculpture. His rippling muscles, flowing hair, and thick beard stand in contrast to his smaller and smooth-limbed sons. The J. Paul Getty Museum, where the sculpture resides today, notes that Foggini is recognized for his “exactitude in anatomical modeling and an equally precise and expert finishing of details.”

After witnessing the excruciating deaths of the priest and his sons, the dazed Trojans reason that the wooden horse was intended as a sacred gift for the gods, and they move this trophy inside the walls of Troy. An elite Greek force sequestered in the horse emerges during the night and opens the gates for the Greeks army, which has stealthily returned. The Greeks overwhelm the unprepared Trojans, sack the city, and win the war. Laocöon had properly understood the ruse and his admonition had proven accurate.

Bad decisions and character flaws were not solely responsible for the fall of Troy. The major Greek deities had their own interests in the outcome, and their many intercessions caused death and mayhem on both sides before the final reckoning. Historian Barbara Tuchman explains, “Taking sides and playing favorites, potent but fickle, conjuring deceptive images, altering the fortunes of battles to suit their desires, whispering, tricking, falsifying, even inducing the Greeks through deceit to continue when they are ready to give up and go home, the gods keep the combatants engaged while heroes die and homelands suffer.”

Had the Trojan leaders conducting their own careful investigation to see whether the Greek forces had actually retreated and to see what was lurking inside the horse, then the Greeks might not have succeeded in executing perhaps the best-known subterfuge described in literature. Foggini’s bronze statue reminds us that the consequences of dismissing admonitions and failing to investigate can be catastrophic.
